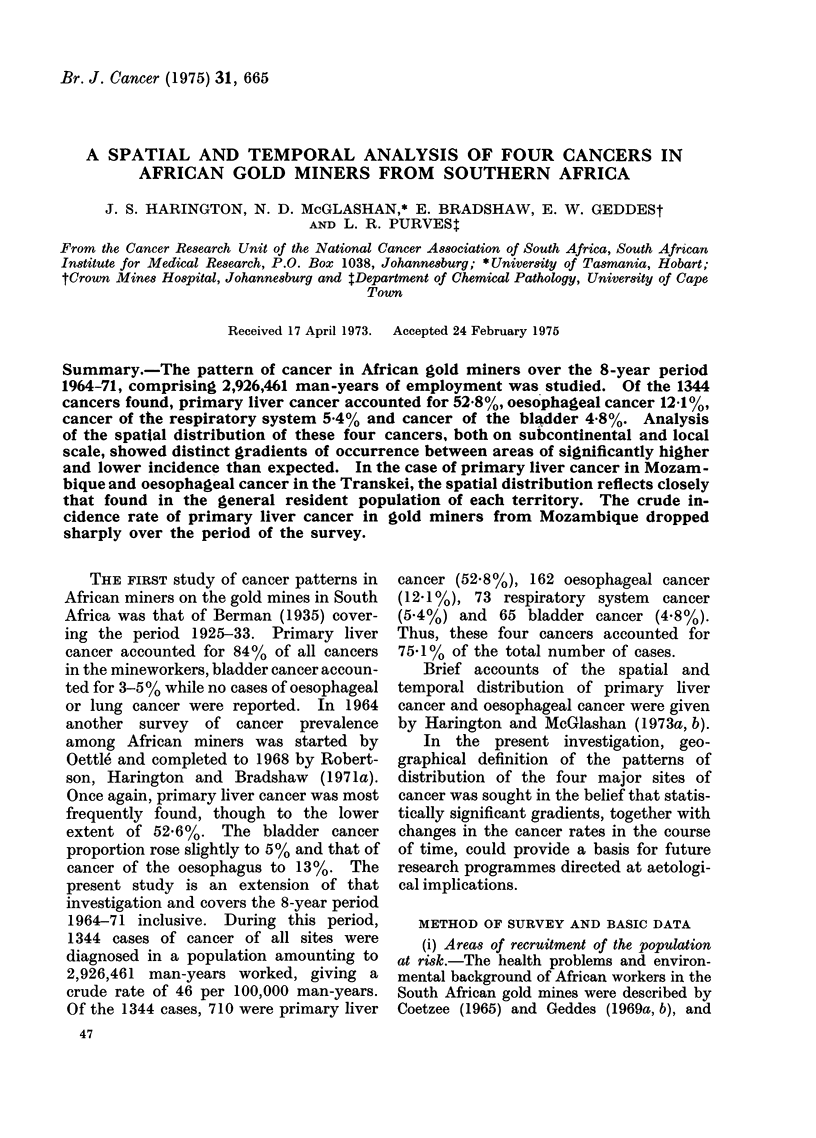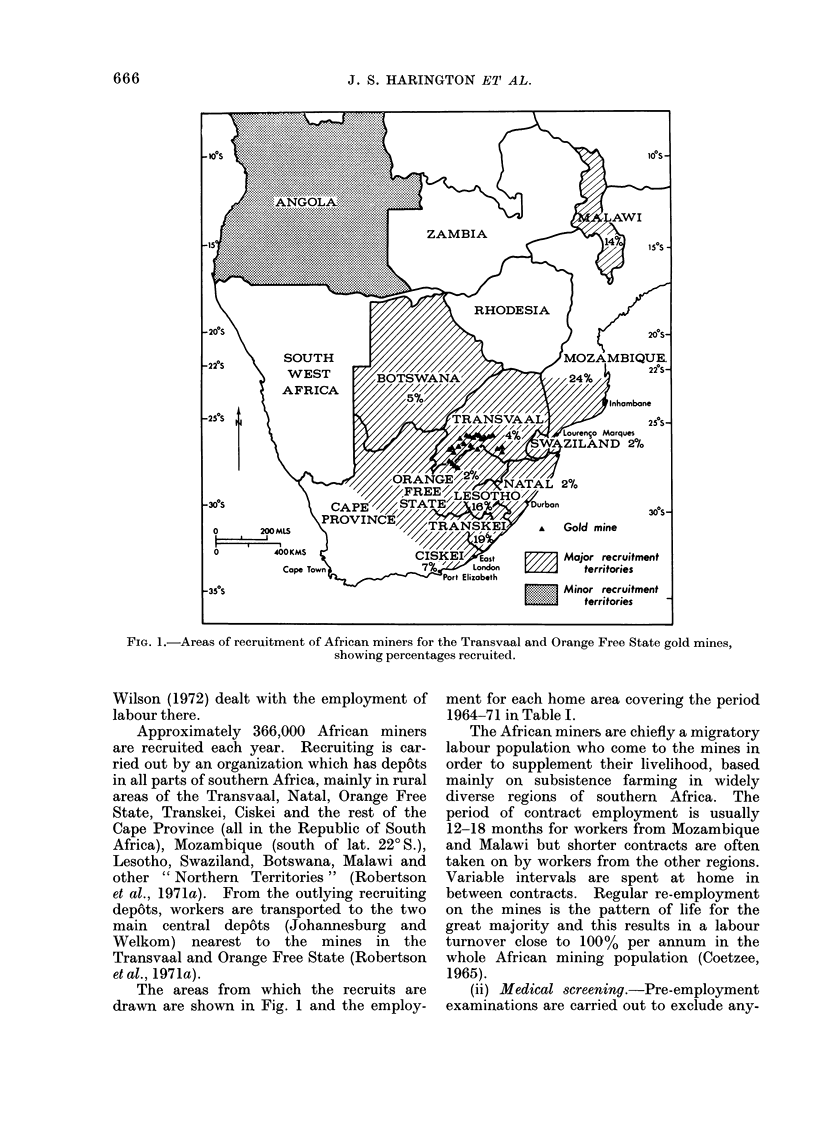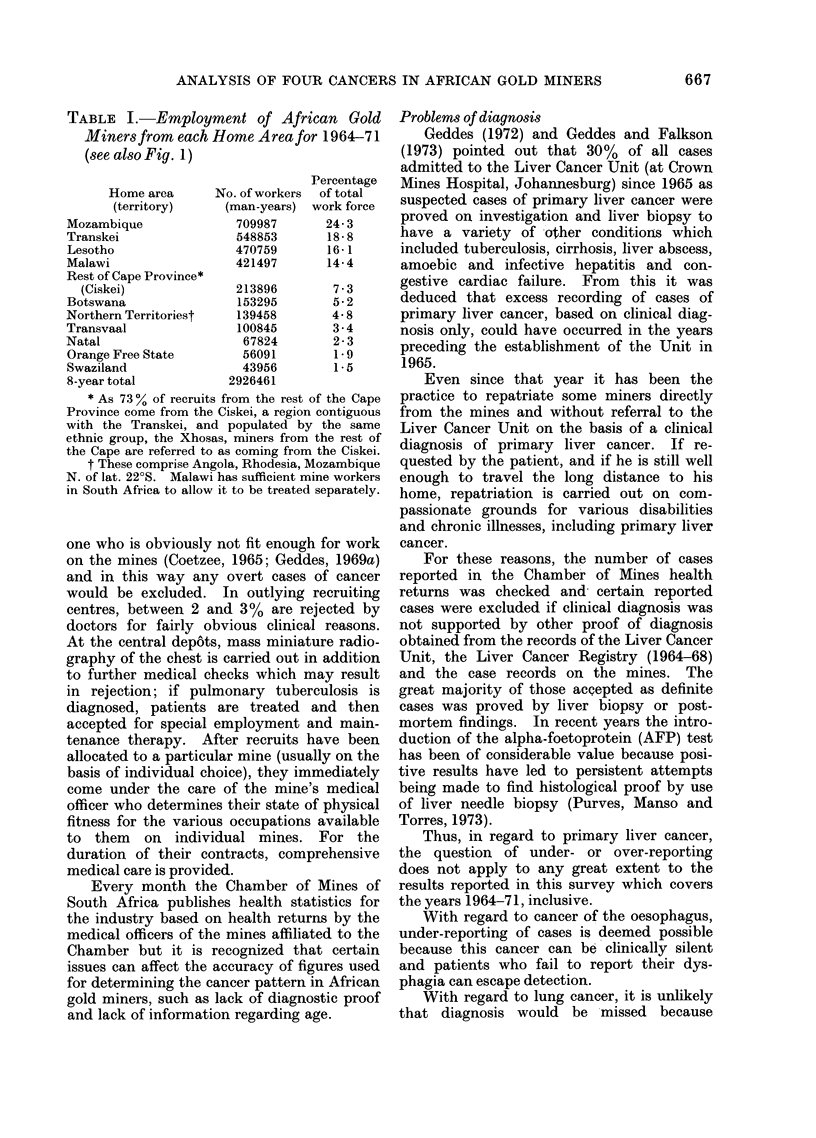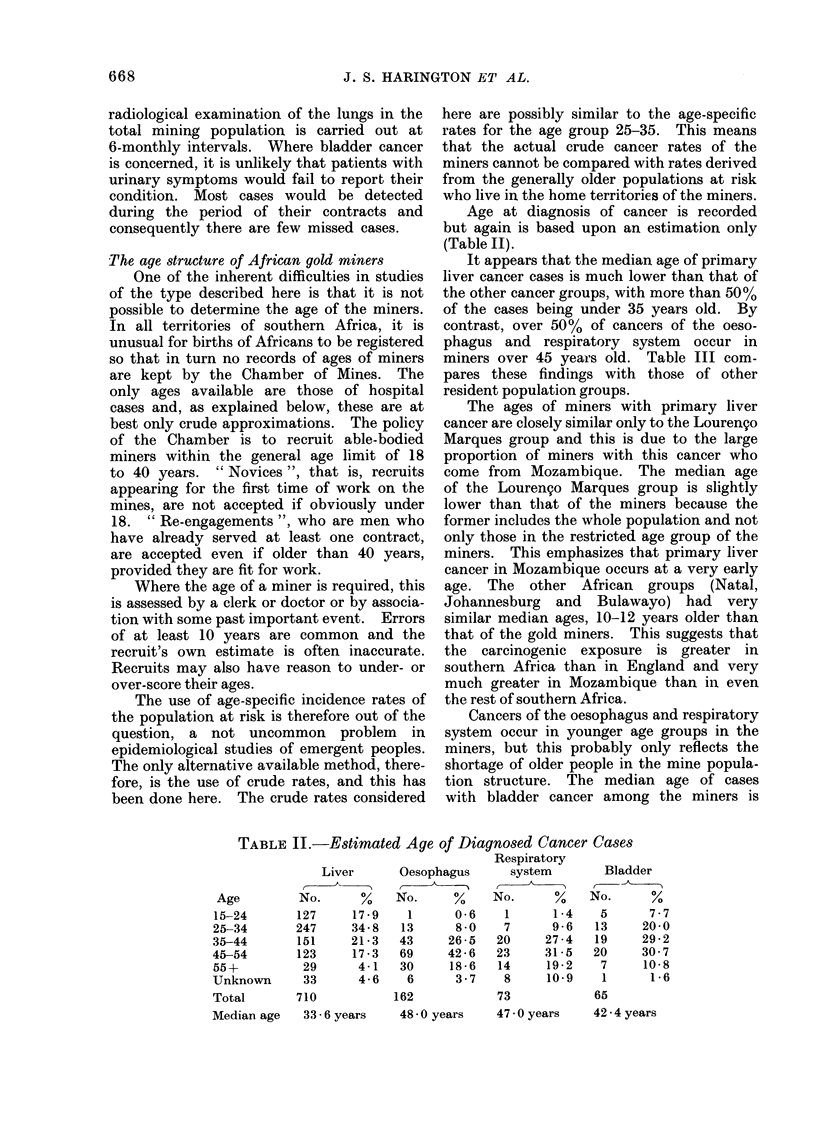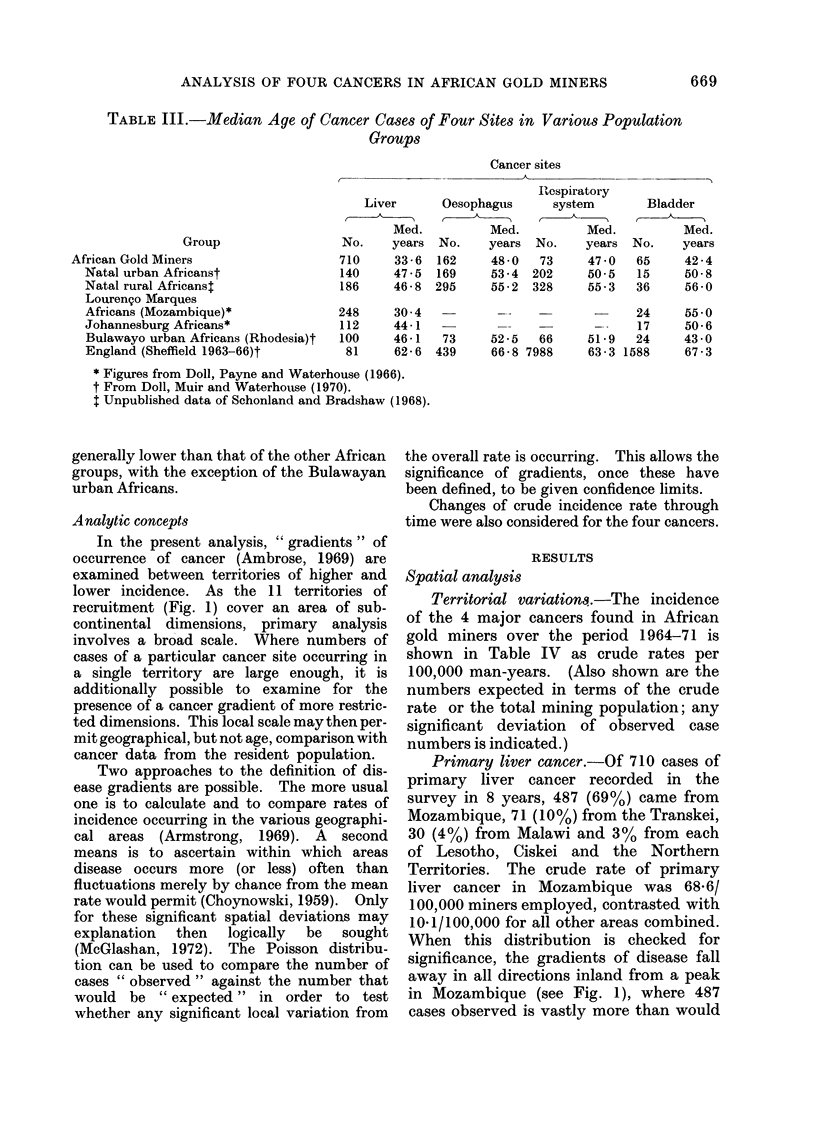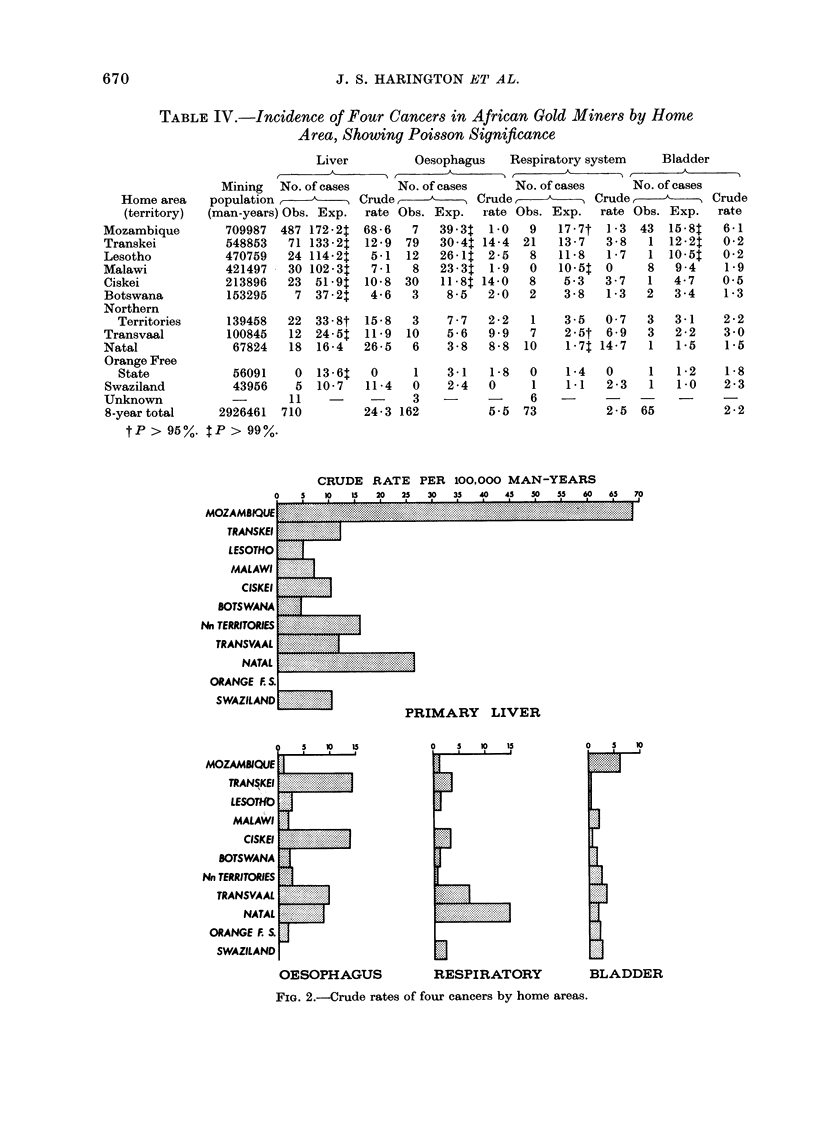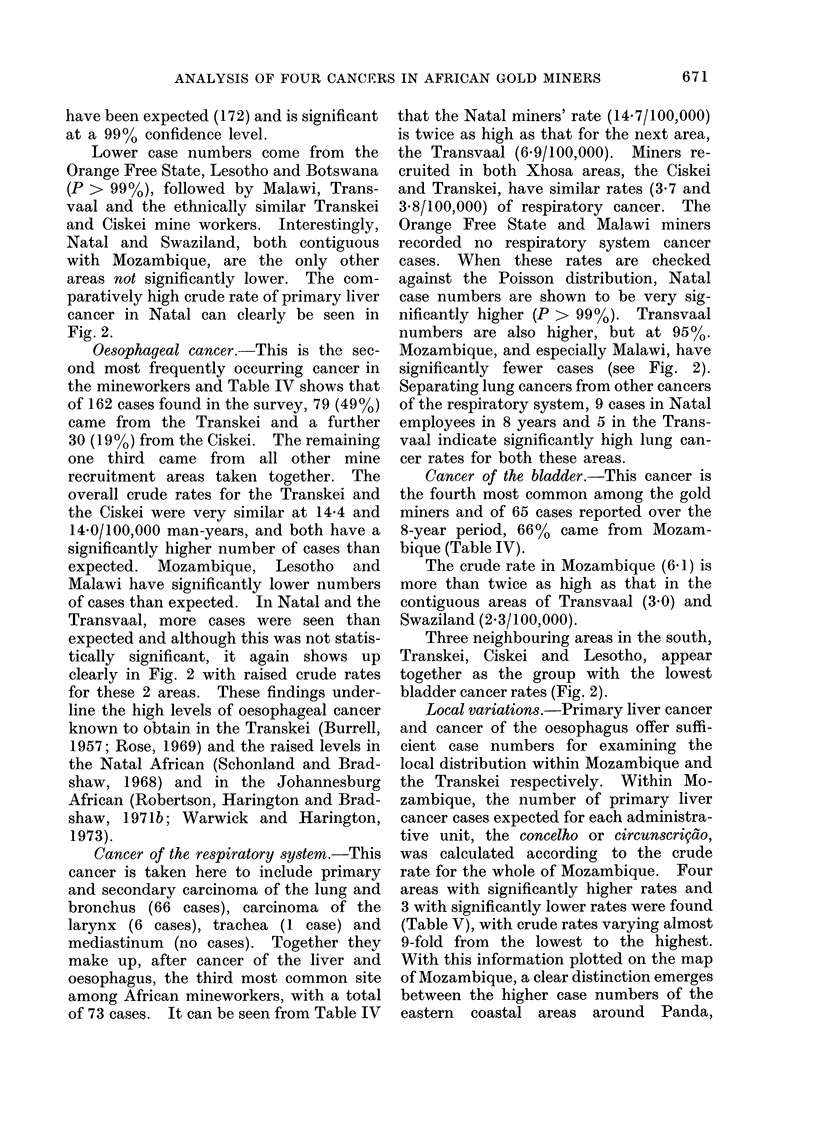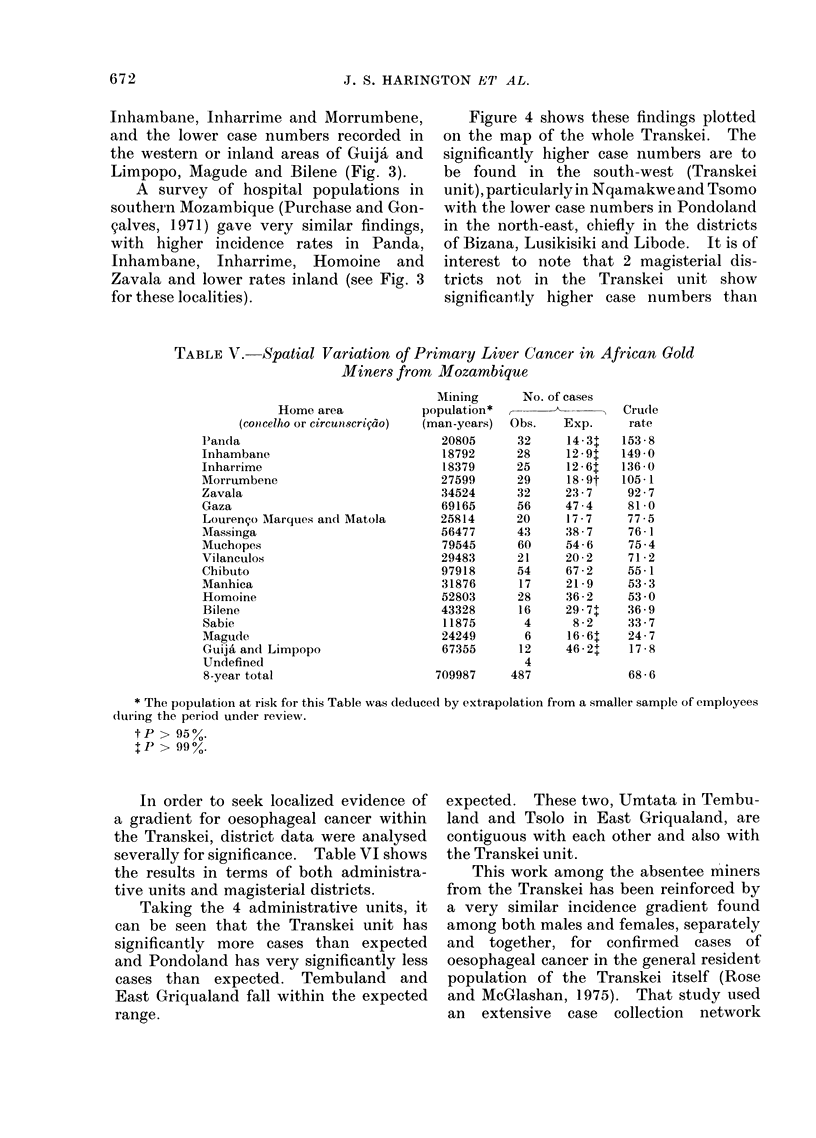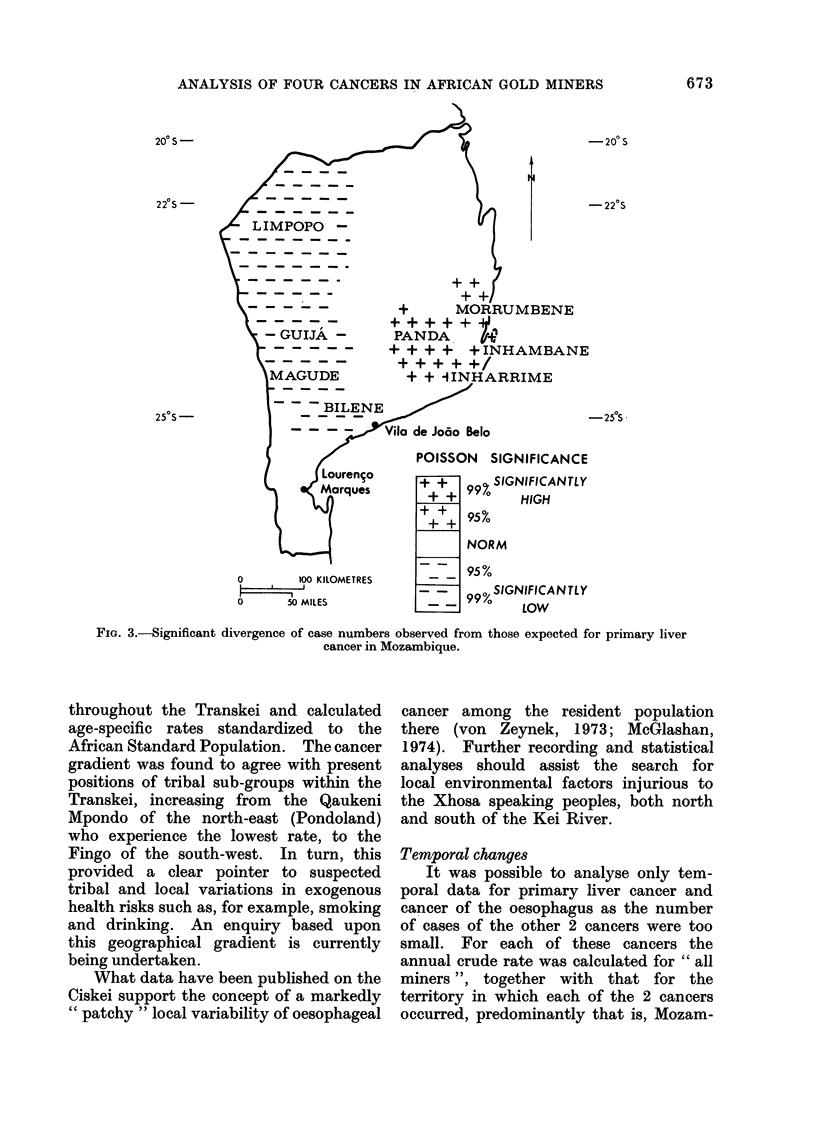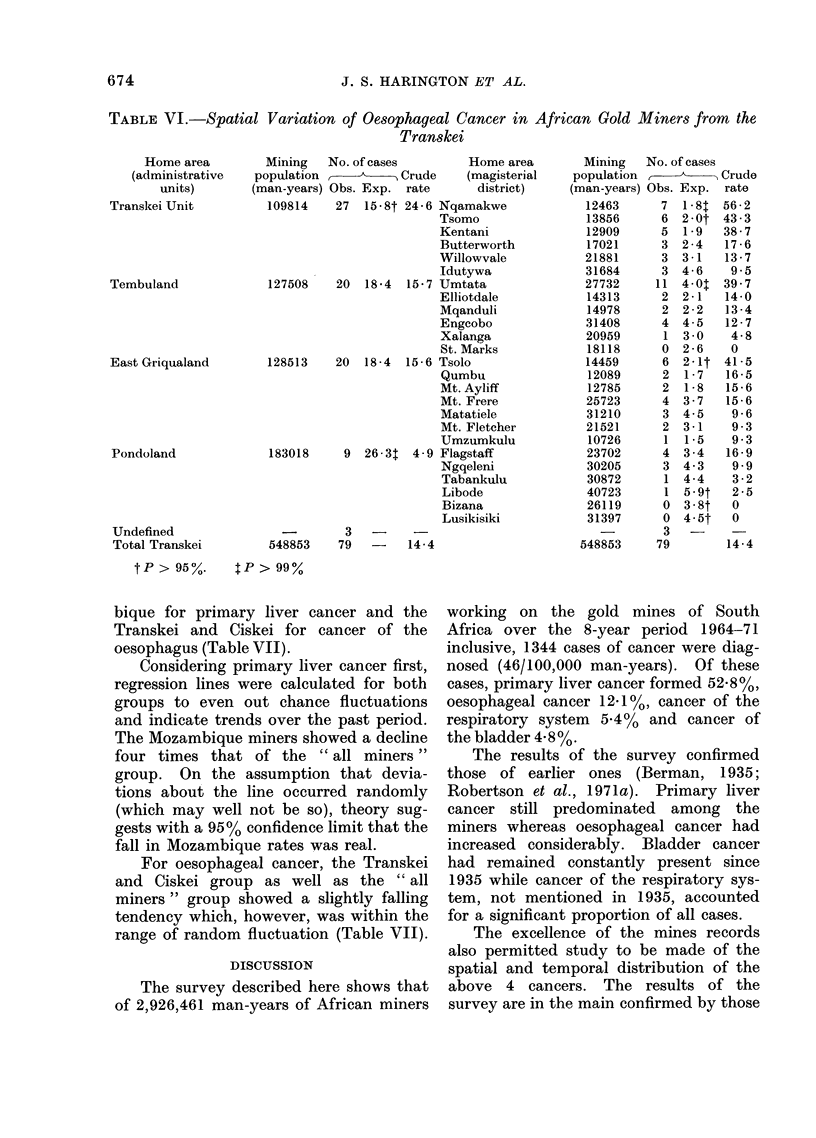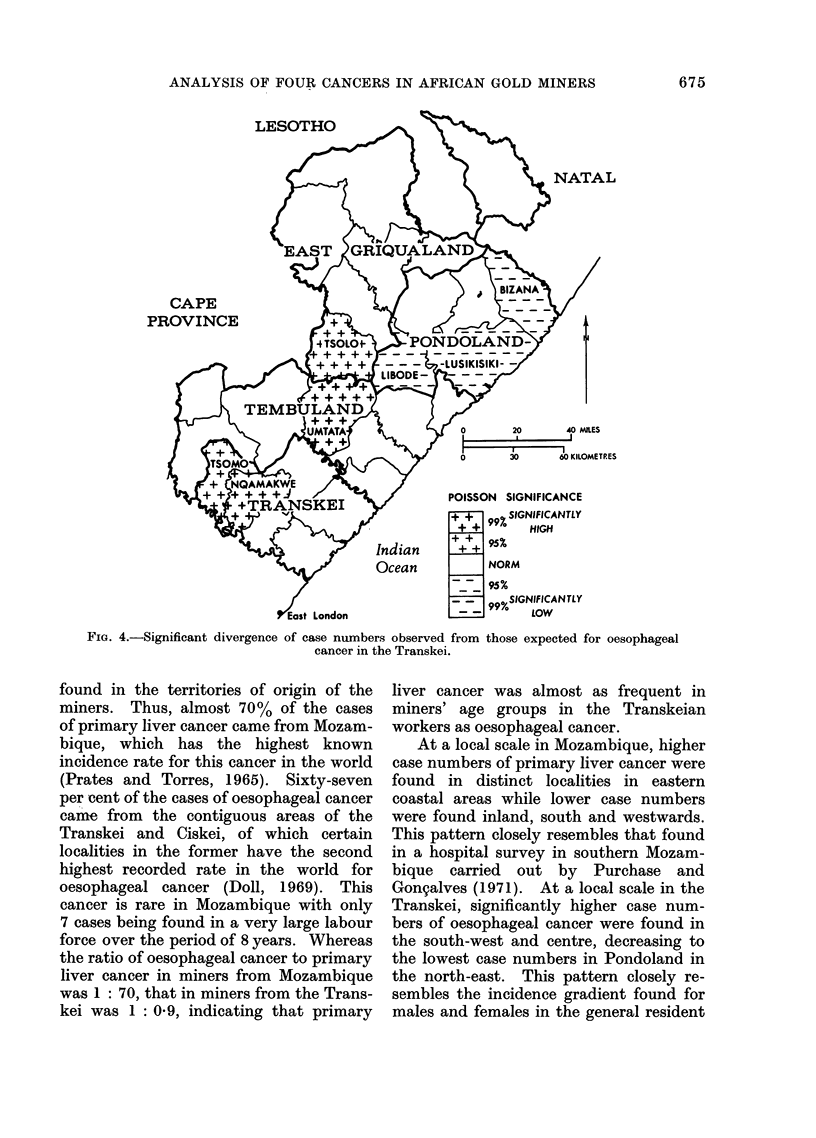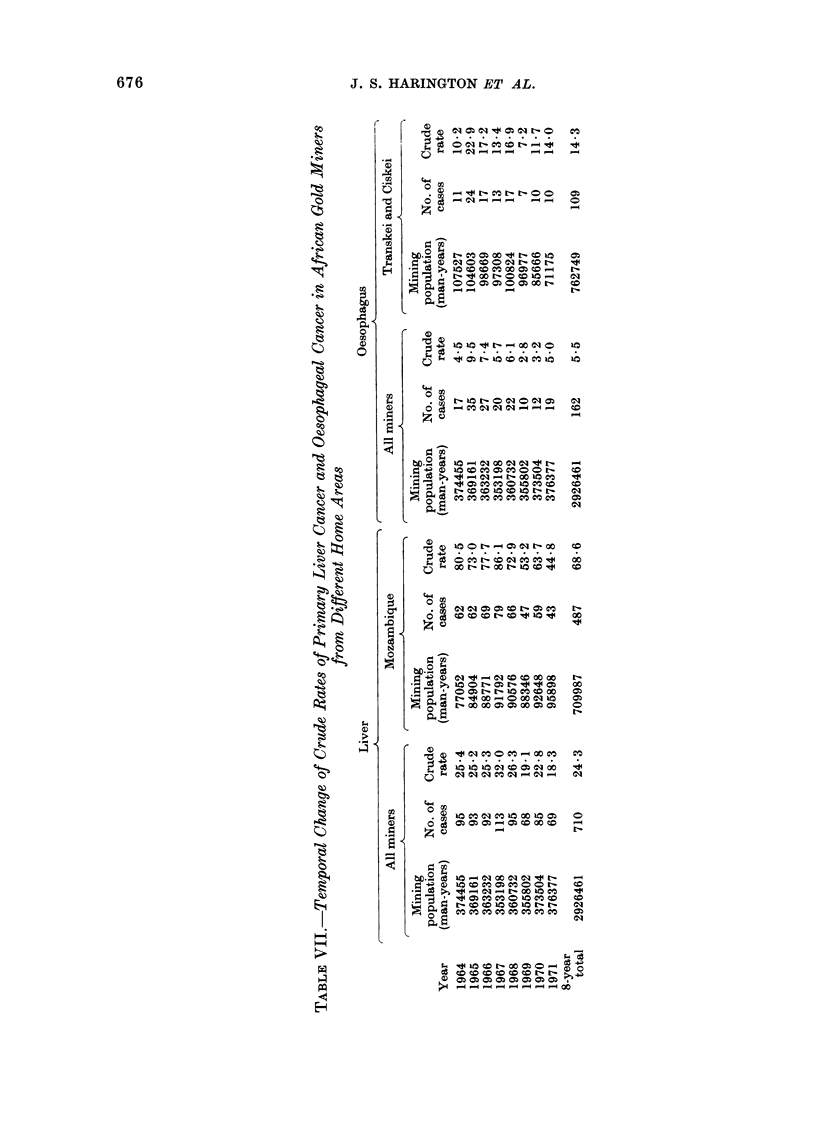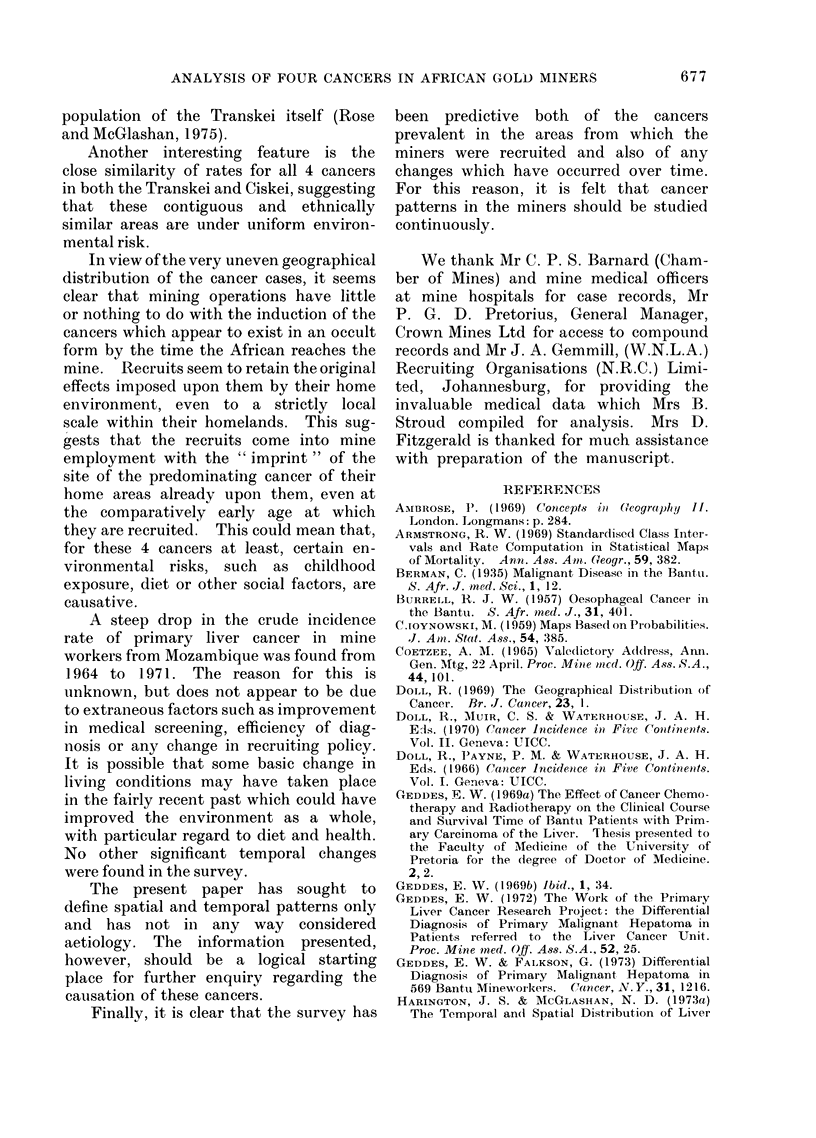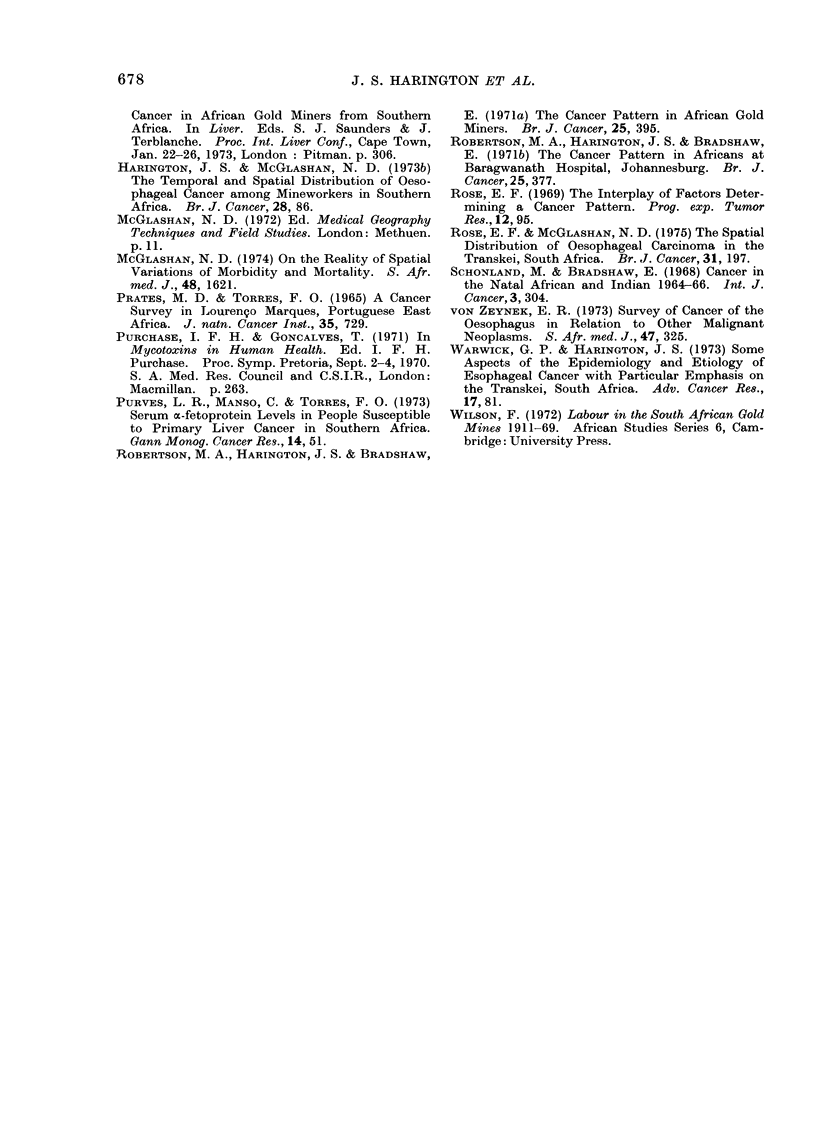# A spatial and temporal analysis of four cancers in African gold miners from Southern Africa.

**DOI:** 10.1038/bjc.1975.114

**Published:** 1975-06

**Authors:** J. S. Harington, N. D. McGlashan, E. Bradshaw, E. W. Geddes, L. R. Purves

## Abstract

The pattern of cancer in African gold miners over the 8-year period 1964-71, comprising 2,926,461 man-years of employment was studied. Of the 1344 cancers found, primary liver cancer accounted for 52-8%, oesophageal cancer 12-1%, cancer of the respiratory system 5-4% and cancer of the bladder 4-8%. Analysis of the spatial distribution of these four cancers, both on subcontinental and local scale, showed distinct gradients of occurrence between areas of significantly higher and lower incidence than expected. In the case of primary liver cancer in Mozambique and oesophageal cancer in the Transkei, the spatial distribution reflects closely that found in the general resident population of each territory. The crude incidence rate of primary liver cancer in gold miners from Mozambique dropped sharply over the period of the survey.


*Br. J. Cancer* (1975) **31**, 665

### A SPATIAL AND TEMPORAL ANALYSIS OF FOUR CANCERS IN AFRICAN GOLD MINERS FROM SOUTHERN AFRICA

J. S. HARINGTON, N. D. McGLASHAN,* E. BRADSHAW, E. W. GEDDES† AND L. R. PURVES‡

*From the Cancer Research Unit of the National Cancer Association of South Africa, South African Institute for Medical Research, P.O. Box 1038, Johannesburg; *University of Tasmania, Hobart; †Crown Mines Hospital, Johannesburg and ‡Department of Chemical Pathology, University of Cape Town*

Received 17 April 1973. Accepted 24 February 1975

**Summary.—The pattern of cancer in African gold miners over the 8-year period 1964–71, comprising 2,926,461 man-years of employment was studied. Of the 1344 cancers found, primary liver cancer accounted for 52·8%, oesophageal cancer 12·1%, cancer of the respiratory system 5·4% and cancer of the bladder 4·8%. Analysis of the spatial distribution of these four cancers, both on subcontinental and local scale, showed distinct gradients of occurrence between areas of significantly higher and lower incidence than expected. In the case of primary liver cancer in Mozambique and oesophageal cancer in the Transkei, the spatial distribution reflects closely that found in the general resident population of each territory. The crude incidence rate of primary liver cancer in gold miners from Mozambique dropped sharply over the period of the survey.**

THE FIRST study of cancer patterns in African miners on the gold mines in South Africa was that of Berman (1935) covering the period 1925–33. Primary liver cancer accounted for 84% of all cancers in the mineworkers, bladder cancer accounted for 3–5% while no cases of oesophageal or lung cancer were reported. In 1964 another survey of cancer prevalence among African miners was started by Oettlé and completed to 1968 by Robertson, Harington and Bradshaw (1971*a*). Once again, primary liver cancer was most frequently found, though to the lower extent of 52·6%. The bladder cancer proportion rose slightly to 5% and that of cancer of the oesophagus to 13%. The present study is an extension of that investigation and covers the 8-year period 1964–71 inclusive. During this period, 1344 cases of cancer of all sites were diagnosed in a population amounting to 2,926,461 man-years worked, giving a crude rate of 46 per 100,000 man-years. Of the 1344 cases, 710 were primary liver cancer (52·8%), 162 oesophageal cancer (12·1%), 73 respiratory system cancer (5·4%) and 65 bladder cancer (4·8%). Thus, these four cancers accounted for 75·1% of the total number of cases.

Brief accounts of the spatial and temporal distribution of primary liver cancer and oesophageal cancer were given by Harington and McGlashan (1973*a*, *b*).

In the present investigation, geographical definition of the patterns of distribution of the four major sites of cancer was sought in the belief that statistically significant gradients, together with changes in the cancer rates in the course of time, could provide a basis for future research programmes directed at aetological implications.

### METHOD OF SURVEY AND BASIC DATA

(i) *Areas of recruitment of the population at risk.*—The health problems and environmental background of African workers in the South African gold mines were described by Coetzee (1965) and Geddes (1969*a*, *b*), and

FIG. 1.—Areas of recruitment of African miners for the Transvaal and Orange Free State gold mines, showing percentages recruited.

Wilson (1972) dealt with the employment of labour there.

Approximately 366,000 African miners are recruited each year. Recruiting is carried out by an organization which has depôts in all parts of southern Africa, mainly in rural areas of the Transvaal, Natal, Orange Free State, Transkei, Ciskei and the rest of the Cape Province (all in the Republic of South Africa), Mozambique (south of lat. 22° S.), Lesotho, Swaziland, Botswana, Malawi and other "Northern Territories" (Robertson *et al.*, 1971*a*). From the outlying recruiting depôts, workers are transported to the two main central depôts (Johannesburg and Welkom) nearest to the mines in the Transvaal and Orange Free State (Robertson *et al.*, 1971*a*).

The areas from which the recruits are drawn are shown in Fig. 1 and the employment for each home area covering the period 1964–71 in Table I.

The African miners are chiefly a migratory labour population who come to the mines in order to supplement their livelihood, based mainly on subsistence farming in widely diverse regions of southern Africa. The period of contract employment is usually 12–18 months for workers from Mozambique and Malawi but shorter contracts are often taken on by workers from the other regions. Variable intervals are spent at home in between contracts. Regular re-employment on the mines is the pattern of life for the great majority and this results in a labour turnover close to 100% per annum in the whole African mining population (Coetzee, 1965).

(ii) *Medical screening.*—Pre-employment examinations are carried out to exclude any-

TABLE I.—*Employment of African Gold Miners from each Home Area for* 1964–71 (*see also Fig.* 1)

| Home area (territory) | No. of workers (man-years) | Percentage of total work force |
|---|---|---|
| Mozambique | 709987 | 24·3 |
| Transkei | 548853 | 18·8 |
| Lesotho | 470759 | 16·1 |
| Malawi | 421497 | 14·4 |
| Rest of Cape Province* (Ciskei) | 213896 | 7·3 |
| Botswana | 153295 | 5·2 |
| Northern Territories† | 139458 | 4·8 |
| Transvaal | 100845 | 3·4 |
| Natal | 67824 | 2·3 |
| Orange Free State | 56091 | 1·9 |
| Swaziland | 43956 | 1·5 |
| 8-year total | 2926461 | |

* As 73% of recruits from the rest of the Cape Province come from the Ciskei, a region contiguous with the Transkei, and populated by the same ethnic group, the Xhosas, miners from the rest of the Cape are referred to as coming from the Ciskei.

† These comprise Angola, Rhodesia, Mozambique N. of lat. 22°S. Malawi has sufficient mine workers in South Africa to allow it to be treated separately.

one who is obviously not fit enough for work on the mines (Coetzee, 1965; Geddes, 1969*a*) and in this way any overt cases of cancer would be excluded. In outlying recruiting centres, between 2 and 3% are rejected by doctors for fairly obvious clinical reasons. At the central depôts, mass miniature radiography of the chest is carried out in addition to further medical checks which may result in rejection; if pulmonary tuberculosis is diagnosed, patients are treated and then accepted for special employment and maintenance therapy. After recruits have been allocated to a particular mine (usually on the basis of individual choice), they immediately come under the care of the mine's medical officer who determines their state of physical fitness for the various occupations available to them on individual mines. For the duration of their contracts, comprehensive medical care is provided.

Every month the Chamber of Mines of South Africa publishes health statistics for the industry based on health returns by the medical officers of the mines affiliated to the Chamber but it is recognized that certain issues can affect the accuracy of figures used for determining the cancer pattern in African gold miners, such as lack of diagnostic proof and lack of information regarding age.

*Problems of diagnosis*

Geddes (1972) and Geddes and Falkson (1973) pointed out that 30% of all cases admitted to the Liver Cancer Unit (at Crown Mines Hospital, Johannesburg) since 1965 as suspected cases of primary liver cancer were proved on investigation and liver biopsy to have a variety of other conditions which included tuberculosis, cirrhosis, liver abscess, amoebic and infective hepatitis and congestive cardiac failure. From this it was deduced that excess recording of cases of primary liver cancer, based on clinical diagnosis only, could have occurred in the years preceding the establishment of the Unit in 1965.

Even since that year it has been the practice to repatriate some miners directly from the mines and without referral to the Liver Cancer Unit on the basis of a clinical diagnosis of primary liver cancer. If requested by the patient, and if he is still well enough to travel the long distance to his home, repatriation is carried out on compassionate grounds for various disabilities and chronic illnesses, including primary liver cancer.

For these reasons, the number of cases reported in the Chamber of Mines health returns was checked and certain reported cases were excluded if clinical diagnosis was not supported by other proof of diagnosis obtained from the records of the Liver Cancer Unit, the Liver Cancer Registry (1964–68) and the case records on the mines. The great majority of those accepted as definite cases was proved by liver biopsy or post-mortem findings. In recent years the introduction of the alpha-foetoprotein (AFP) test has been of considerable value because positive results have led to persistent attempts being made to find histological proof by use of liver needle biopsy (Purves, Manso and Torres, 1973).

Thus, in regard to primary liver cancer, the question of under- or over-reporting does not apply to any great extent to the results reported in this survey which covers the years 1964–71, inclusive.

With regard to cancer of the oesophagus, under-reporting of cases is deemed possible because this cancer can be clinically silent and patients who fail to report their dysphagia can escape detection.

With regard to lung cancer, it is unlikely that diagnosis would be missed because radiological examination of the lungs in the total mining population is carried out at 6-monthly intervals. Where bladder cancer is concerned, it is unlikely that patients with urinary symptoms would fail to report their condition. Most cases would be detected during the period of their contracts and consequently there are few missed cases.

*The age structure of African gold miners*

One of the inherent difficulties in studies of the type described here is that it is not possible to determine the age of the miners. In all territories of southern Africa, it is unusual for births of Africans to be registered so that in turn no records of ages of miners are kept by the Chamber of Mines. The only ages available are those of hospital cases and, as explained below, these are at best only crude approximations. The policy of the Chamber is to recruit able-bodied miners within the general age limit of 18 to 40 years. " Novices ", that is, recruits appearing for the first time of work on the mines, are not accepted if obviously under 18. " Re-engagements ", who are men who have already served at least one contract, are accepted even if older than 40 years, provided they are fit for work.

Where the age of a miner is required, this is assessed by a clerk or doctor or by association with some past important event. Errors of at least 10 years are common and the recruit's own estimate is often inaccurate. Recruits may also have reason to under- or over-score their ages.

The use of age-specific incidence rates of the population at risk is therefore out of the question, a not uncommon problem in epidemiological studies of emergent peoples. The only alternative available method, therefore, is the use of crude rates, and this has been done here. The crude rates considered here are possibly similar to the age-specific rates for the age group 25–35. This means that the actual crude cancer rates of the miners cannot be compared with rates derived from the generally older populations at risk who live in the home territories of the miners.

Age at diagnosis of cancer is recorded but again is based upon an estimation only (Table II).

It appears that the median age of primary liver cancer cases is much lower than that of the other cancer groups, with more than 50% of the cases being under 35 years old. By contrast, over 50% of cancers of the oesophagus and respiratory system occur in miners over 45 years old. Table III compares these findings with those of other resident population groups.

The ages of miners with primary liver cancer are closely similar only to the Lourenço Marques group and this is due to the large proportion of miners with this cancer who come from Mozambique. The median age of the Lourenço Marques group is slightly lower than that of the miners because the former includes the whole population and not only those in the restricted age group of the miners. This emphasizes that primary liver cancer in Mozambique occurs at a very early age. The other African groups (Natal, Johannesburg and Bulawayo) had very similar median ages, 10–12 years older than that of the gold miners. This suggests that the carcinogenic exposure is greater in southern Africa than in England and very much greater in Mozambique than in even the rest of southern Africa.

Cancers of the oesophagus and respiratory system occur in younger age groups in the miners, but this probably only reflects the shortage of older people in the mine population structure. The median age of cases with bladder cancer among the miners is

TABLE II.—*Estimated Age of Diagnosed Cancer Cases*

| | Liver | | Oesophagus | | Respiratory system | | Bladder | |
|---|---|---|---|---|---|---|---|---|
| Age | No. | % | No. | % | No. | % | No. | % |
| 15–24 | 127 | 17·9 | 1 | 0·6 | 1 | 1·4 | 5 | 7·7 |
| 25–34 | 247 | 34·8 | 13 | 8·0 | 7 | 9·6 | 13 | 20·0 |
| 35–44 | 151 | 21·3 | 43 | 26·5 | 20 | 27·4 | 19 | 29·2 |
| 45–54 | 123 | 17·3 | 69 | 42·6 | 23 | 31·5 | 20 | 30·7 |
| 55+ | 29 | 4·1 | 30 | 18·6 | 14 | 19·2 | 7 | 10·8 |
| Unknown | 33 | 4·6 | 6 | 3·7 | 8 | 10·9 | 1 | 1·6 |
| Total | 710 | | 162 | | 73 | | 65 | |
| Median age | 33·6 years | | 48·0 years | | 47·0 years | | 42·4 years | |

TABLE III.—*Median Age of Cancer Cases of Four Sites in Various Population Groups*

| Group | Liver No. | Liver Med. years | Oesophagus No. | Oesophagus Med. years | Respiratory system No. | Respiratory system Med. years | Bladder No. | Bladder Med. years |
|---|---|---|---|---|---|---|---|---|
| African Gold Miners | 710 | 33·6 | 162 | 48·0 | 73 | 47·0 | 65 | 42·4 |
| Natal urban Africans† | 140 | 47·5 | 169 | 53·4 | 202 | 50·5 | 15 | 50·8 |
| Natal rural Africans‡ | 186 | 46·8 | 295 | 55·2 | 328 | 55·3 | 36 | 56·0 |
| Lourenço Marques Africans (Mozambique)* | 248 | 30·4 | — | — | — | — | 24 | 55·0 |
| Johannesburg Africans* | 112 | 44·1 | — | — | — | — | 17 | 50·6 |
| Bulawayo urban Africans (Rhodesia)† | 100 | 46·1 | 73 | 52·5 | 66 | 51·9 | 24 | 43·0 |
| England (Sheffield 1963–66)† | 81 | 62·6 | 439 | 66·8 | 7988 | 63·3 | 1588 | 67·3 |

* Figures from Doll, Payne and Waterhouse (1966).
† From Doll, Muir and Waterhouse (1970).
‡ Unpublished data of Schonland and Bradshaw (1968).

generally lower than that of the other African groups, with the exception of the Bulawayan urban Africans.

### *Analytic concepts*

In the present analysis, " gradients " of occurrence of cancer (Ambrose, 1969) are examined between territories of higher and lower incidence. As the 11 territories of recruitment (Fig. 1) cover an area of sub-continental dimensions, primary analysis involves a broad scale. Where numbers of cases of a particular cancer site occurring in a single territory are large enough, it is additionally possible to examine for the presence of a cancer gradient of more restricted dimensions. This local scale may then permit geographical, but not age, comparison with cancer data from the resident population.

Two approaches to the definition of disease gradients are possible. The more usual one is to calculate and to compare rates of incidence occurring in the various geographical areas (Armstrong, 1969). A second means is to ascertain within which areas disease occurs more (or less) often than fluctuations merely by chance from the mean rate would permit (Choynowski, 1959). Only for these significant spatial deviations may explanation then logically be sought (McGlashan, 1972). The Poisson distribution can be used to compare the number of cases " observed " against the number that would be " expected " in order to test whether any significant local variation from the overall rate is occurring. This allows the significance of gradients, once these have been defined, to be given confidence limits.

Changes of crude incidence rate through time were also considered for the four cancers.

### RESULTS

### *Spatial analysis*

*Territorial variations*.—The incidence of the 4 major cancers found in African gold miners over the period 1964–71 is shown in Table IV as crude rates per 100,000 man-years. (Also shown are the numbers expected in terms of the crude rate or the total mining population; any significant deviation of observed case numbers is indicated.)

*Primary liver cancer*.—Of 710 cases of primary liver cancer recorded in the survey in 8 years, 487 (69%) came from Mozambique, 71 (10%) from the Transkei, 30 (4%) from Malawi and 3% from each of Lesotho, Ciskei and the Northern Territories. The crude rate of primary liver cancer in Mozambique was 68·6/100,000 miners employed, contrasted with 10·1/100,000 for all other areas combined. When this distribution is checked for significance, the gradients of disease fall away in all directions inland from a peak in Mozambique (see Fig. 1), where 487 cases observed is vastly more than would

TABLE IV.—*Incidence of Four Cancers in African Gold Miners by Home Area, Showing Poisson Significance*

| Home area (territory) | Mining population (man-years) | Liver: No. of cases Obs. | Liver: No. of cases Exp. | Liver: Crude rate | Oesophagus: No. of cases Obs. | Oesophagus: No. of cases Exp. | Oesophagus: Crude rate | Respiratory system: No. of cases Obs. | Respiratory system: No. of cases Exp. | Respiratory system: Crude rate | Bladder: No. of cases Obs. | Bladder: No. of cases Exp. | Bladder: Crude rate |
|---|---|---|---|---|---|---|---|---|---|---|---|---|---|
| Mozambique | 709987 | 487 | 172·2‡ | 68·6 | 7 | 39·3‡ | 1·0 | 9 | 17·7† | 1·3 | 43 | 15·8‡ | 6·1 |
| Transkei | 548853 | 71 | 133·2‡ | 12·9 | 79 | 30·4‡ | 14·4 | 21 | 13·7 | 3·8 | 1 | 12·2‡ | 0·2 |
| Lesotho | 470759 | 24 | 114·2‡ | 5·1 | 12 | 26·1‡ | 2·5 | 8 | 11·8 | 1·7 | 1 | 10·5‡ | 0·2 |
| Malawi | 421497 | 30 | 102·3‡ | 7·1 | 8 | 23·3‡ | 1·9 | 0 | 10·5‡ | 0 | 8 | 9·4 | 1·9 |
| Ciskei | 213896 | 23 | 51·9‡ | 10·8 | 30 | 11·8‡ | 14·0 | 8 | 5·3 | 3·7 | 1 | 4·7 | 0·5 |
| Botswana | 153295 | 7 | 37·2‡ | 4·6 | 3 | 8·5 | 2·0 | 2 | 3·8 | 1·3 | 2 | 3·4 | 1·3 |
| Northern Territories | 139458 | 22 | 33·8† | 15·8 | 3 | 7·7 | 2·2 | 1 | 3·5 | 0·7 | 3 | 3·1 | 2·2 |
| Transvaal | 100845 | 12 | 24·5‡ | 11·9 | 10 | 5·6 | 9·9 | 7 | 2·5† | 6·9 | 3 | 2·2 | 3·0 |
| Natal | 67824 | 18 | 16·4 | 26·5 | 6 | 3·8 | 8·8 | 10 | 1·7‡ | 14·7 | 1 | 1·5 | 1·5 |
| Orange Free State | 56091 | 0 | 13·6‡ | 0 | 1 | 3·1 | 1·8 | 0 | 1·4 | 0 | 1 | 1·2 | 1·8 |
| Swaziland | 43956 | 5 | 10·7 | 11·4 | 0 | 2·4 | 0 | 1 | 1·1 | 2·3 | 1 | 1·0 | 2·3 |
| Unknown | — | 11 | — | — | 3 | — | — | 6 | — | — | — | — | — |
| 8-year total | 2926461 | 710 | | 24·3 | 162 | | 5·5 | 73 | | 2·5 | 65 | | 2·2 |

† $P > 95\%$. ‡ $P > 99\%$.

FIG. 2.—Crude rates of four cancers by home areas.

have been expected (172) and is significant at a 99% confidence level.

Lower case numbers come from the Orange Free State, Lesotho and Botswana ($P > 99\%$), followed by Malawi, Transvaal and the ethnically similar Transkei and Ciskei mine workers. Interestingly, Natal and Swaziland, both contiguous with Mozambique, are the only other areas *not* significantly lower. The comparatively high crude rate of primary liver cancer in Natal can clearly be seen in Fig. 2.

*Oesophageal cancer.*—This is the second most frequently occurring cancer in the mineworkers and Table IV shows that of 162 cases found in the survey, 79 (49%) came from the Transkei and a further 30 (19%) from the Ciskei. The remaining one third came from all other mine recruitment areas taken together. The overall crude rates for the Transkei and the Ciskei were very similar at 14·4 and 14·0/100,000 man-years, and both have a significantly higher number of cases than expected. Mozambique, Lesotho and Malawi have significantly lower numbers of cases than expected. In Natal and the Transvaal, more cases were seen than expected and although this was not statistically significant, it again shows up clearly in Fig. 2 with raised crude rates for these 2 areas. These findings underline the high levels of oesophageal cancer known to obtain in the Transkei (Burrell, 1957; Rose, 1969) and the raised levels in the Natal African (Schonland and Bradshaw, 1968) and in the Johannesburg African (Robertson, Harington and Bradshaw, 1971*b*; Warwick and Harington, 1973).

*Cancer of the respiratory system.*—This cancer is taken here to include primary and secondary carcinoma of the lung and bronchus (66 cases), carcinoma of the larynx (6 cases), trachea (1 case) and mediastinum (no cases). Together they make up, after cancer of the liver and oesophagus, the third most common site among African mineworkers, with a total of 73 cases. It can be seen from Table IV that the Natal miners' rate (14·7/100,000) is twice as high as that for the next area, the Transvaal (6·9/100,000). Miners recruited in both Xhosa areas, the Ciskei and Transkei, have similar rates (3·7 and 3·8/100,000) of respiratory cancer. The Orange Free State and Malawi miners recorded no respiratory system cancer cases. When these rates are checked against the Poisson distribution, Natal case numbers are shown to be very significantly higher ($P > 99\%$). Transvaal numbers are also higher, but at 95%. Mozambique, and especially Malawi, have significantly fewer cases (see Fig. 2). Separating lung cancers from other cancers of the respiratory system, 9 cases in Natal employees in 8 years and 5 in the Transvaal indicate significantly high lung cancer rates for both these areas.

*Cancer of the bladder.*—This cancer is the fourth most common among the gold miners and of 65 cases reported over the 8-year period, 66% came from Mozambique (Table IV).

The crude rate in Mozambique (6·1) is more than twice as high as that in the contiguous areas of Transvaal (3·0) and Swaziland (2·3/100,000).

Three neighbouring areas in the south, Transkei, Ciskei and Lesotho, appear together as the group with the lowest bladder cancer rates (Fig. 2).

*Local variations.*—Primary liver cancer and cancer of the oesophagus offer sufficient case numbers for examining the local distribution within Mozambique and the Transkei respectively. Within Mozambique, the number of primary liver cancer cases expected for each administrative unit, the *concelho* or *circunscrição*, was calculated according to the crude rate for the whole of Mozambique. Four areas with significantly higher rates and 3 with significantly lower rates were found (Table V), with crude rates varying almost 9-fold from the lowest to the highest. With this information plotted on the map of Mozambique, a clear distinction emerges between the higher case numbers of the eastern coastal areas around Panda,

Inhambane, Inharrime and Morrumbene, and the lower case numbers recorded in the western or inland areas of Guijá and Limpopo, Magude and Bilene (Fig. 3).

A survey of hospital populations in southern Mozambique (Purchase and Gonçalves, 1971) gave very similar findings, with higher incidence rates in Panda, Inhambane, Inharrime, Homoine and Zavala and lower rates inland (see Fig. 3 for these localities).

Figure 4 shows these findings plotted on the map of the whole Transkei. The significantly higher case numbers are to be found in the south-west (Transkei unit), particularly in Nqamakwe and Tsomo with the lower case numbers in Pondoland in the north-east, chiefly in the districts of Bizana, Lusikisiki and Libode. It is of interest to note that 2 magisterial districts not in the Transkei unit show significantly higher case numbers than expected. These two, Umtata in Tembuland and Tsolo in East Griqualand, are contiguous with each other and also with the Transkei unit.

TABLE V.—*Spatial Variation of Primary Liver Cancer in African Gold Miners from Mozambique*

| Home area (*concelho* or *circunscrição*) | Mining population* (man-years) | No. of cases Obs. | No. of cases Exp. | Crude rate |
|---|---|---|---|---|
| Panda | 20805 | 32 | 14·3‡ | 153·8 |
| Inhambane | 18792 | 28 | 12·9‡ | 149·0 |
| Inharrime | 18379 | 25 | 12·6‡ | 136·0 |
| Morrumbene | 27599 | 29 | 18·9† | 105·1 |
| Zavala | 34524 | 32 | 23·7 | 92·7 |
| Gaza | 69165 | 56 | 47·4 | 81·0 |
| Lourenço Marques and Matola | 25814 | 20 | 17·7 | 77·5 |
| Massinga | 56477 | 43 | 38·7 | 76·1 |
| Muchopes | 79545 | 60 | 54·6 | 75·4 |
| Vilanculos | 29483 | 21 | 20·2 | 71·2 |
| Chibuto | 97918 | 54 | 67·2 | 55·1 |
| Manhica | 31876 | 17 | 21·9 | 53·3 |
| Homoine | 52803 | 28 | 36·2 | 53·0 |
| Bilene | 43328 | 16 | 29·7‡ | 36·9 |
| Sabie | 11875 | 4 | 8·2 | 33·7 |
| Magude | 24249 | 6 | 16·6‡ | 24·7 |
| Guijá and Limpopo | 67355 | 12 | 46·2‡ | 17·8 |
| Undefined | | 4 | | |
| 8-year total | 709987 | 487 | | 68·6 |

* The population at risk for this Table was deduced by extrapolation from a smaller sample of employees during the period under review.

† $P > 95\%$.

‡ $P > 99\%$.

In order to seek localized evidence of a gradient for oesophageal cancer within the Transkei, district data were analysed severally for significance. Table VI shows the results in terms of both administrative units and magisterial districts.

Taking the 4 administrative units, it can be seen that the Transkei unit has significantly more cases than expected and Pondoland has very significantly less cases than expected. Tembuland and East Griqualand fall within the expected range.

This work among the absentee miners from the Transkei has been reinforced by a very similar incidence gradient found among both males and females, separately and together, for confirmed cases of oesophageal cancer in the general resident population of the Transkei itself (Rose and McGlashan, 1975). That study used an extensive case collection network

FIG. 3.—Significant divergence of case numbers observed from those expected for primary liver cancer in Mozambique.

throughout the Transkei and calculated age-specific rates standardized to the African Standard Population. The cancer gradient was found to agree with present positions of tribal sub-groups within the Transkei, increasing from the Qaukeni Mpondo of the north-east (Pondoland) who experience the lowest rate, to the Fingo of the south-west. In turn, this provided a clear pointer to suspected tribal and local variations in exogenous health risks such as, for example, smoking and drinking. An enquiry based upon this geographical gradient is currently being undertaken.

What data have been published on the Ciskei support the concept of a markedly " patchy " local variability of oesophageal cancer among the resident population there (von Zeynek, 1973; McGlashan, 1974). Further recording and statistical analyses should assist the search for local environmental factors injurious to the Xhosa speaking peoples, both north and south of the Kei River.

*Temporal changes*

It was possible to analyse only temporal data for primary liver cancer and cancer of the oesophagus as the number of cases of the other 2 cancers were too small. For each of these cancers the annual crude rate was calculated for " all miners ", together with that for the territory in which each of the 2 cancers occurred, predominantly that is, Mozam-

TABLE VI.—*Spatial Variation of Oesophageal Cancer in African Gold Miners from the Transkei*

| Home area (administrative units) | Mining population (man-years) | No. of cases Obs. | Exp. | Crude rate | Home area (magisterial district) | Mining population (man-years) | No. of cases Obs. | Exp. | Crude rate |
|---|---|---|---|---|---|---|---|---|---|
| Transkei Unit | 109814 | 27 | 15·8† | 24·6 | Nqamakwe | 12463 | 7 | 1·8‡ | 56·2 |
| | | | | | Tsomo | 13856 | 6 | 2·0† | 43·3 |
| | | | | | Kentani | 12909 | 5 | 1·9 | 38·7 |
| | | | | | Butterworth | 17021 | 3 | 2·4 | 17·6 |
| | | | | | Willowvale | 21881 | 3 | 3·1 | 13·7 |
| | | | | | Idutywa | 31684 | 3 | 4·6 | 9·5 |
| Tembuland | 127508 | 20 | 18·4 | 15·7 | Umtata | 27732 | 11 | 4·0‡ | 39·7 |
| | | | | | Elliotdale | 14313 | 2 | 2·1 | 14·0 |
| | | | | | Mqanduli | 14978 | 2 | 2·2 | 13·4 |
| | | | | | Engcobo | 31408 | 4 | 4·5 | 12·7 |
| | | | | | Xalanga | 20959 | 1 | 3·0 | 4·8 |
| | | | | | St. Marks | 18118 | 0 | 2·6 | 0 |
| East Griqualand | 128513 | 20 | 18·4 | 15·6 | Tsolo | 14459 | 6 | 2·1† | 41·5 |
| | | | | | Qumbu | 12089 | 2 | 1·7 | 16·5 |
| | | | | | Mt. Ayliff | 12785 | 2 | 1·8 | 15·6 |
| | | | | | Mt. Frere | 25723 | 4 | 3·7 | 15·6 |
| | | | | | Matatiele | 31210 | 3 | 4·5 | 9·6 |
| | | | | | Mt. Fletcher | 21521 | 2 | 3·1 | 9·3 |
| | | | | | Umzumkulu | 10726 | 1 | 1·5 | 9·3 |
| Pondoland | 183018 | 9 | 26·3‡ | 4·9 | Flagstaff | 23702 | 4 | 3·4 | 16·9 |
| | | | | | Ngqeleni | 30205 | 3 | 4·3 | 9·9 |
| | | | | | Tabankulu | 30872 | 1 | 4·4 | 3·2 |
| | | | | | Libode | 40723 | 1 | 5·9† | 2·5 |
| | | | | | Bizana | 26119 | 0 | 3·8† | 0 |
| | | | | | Lusikisiki | 31397 | 0 | 4·5† | 0 |
| Undefined | — | 3 | — | — | | — | 3 | — | — |
| Total Transkei | 548853 | 79 | — | 14·4 | | 548853 | 79 | | 14·4 |

† $P > 95\%$. ‡ $P > 99\%$

bique for primary liver cancer and the Transkei and Ciskei for cancer of the oesophagus (Table VII).

Considering primary liver cancer first, regression lines were calculated for both groups to even out chance fluctuations and indicate trends over the past period. The Mozambique miners showed a decline four times that of the " all miners " group. On the assumption that deviations about the line occurred randomly (which may well not be so), theory suggests with a 95% confidence limit that the fall in Mozambique rates was real.

For oesophageal cancer, the Transkei and Ciskei group as well as the " all miners " group showed a slightly falling tendency which, however, was within the range of random fluctuation (Table VII).

### DISCUSSION

The survey described here shows that of 2,926,461 man-years of African miners working on the gold mines of South Africa over the 8-year period 1964–71 inclusive, 1344 cases of cancer were diagnosed (46/100,000 man-years). Of these cases, primary liver cancer formed 52·8%, oesophageal cancer 12·1%, cancer of the respiratory system 5·4% and cancer of the bladder 4·8%.

The results of the survey confirmed those of earlier ones (Berman, 1935; Robertson *et al.*, 1971*a*). Primary liver cancer still predominated among the miners whereas oesophageal cancer had increased considerably. Bladder cancer had remained constantly present since 1935 while cancer of the respiratory system, not mentioned in 1935, accounted for a significant proportion of all cases.

The excellence of the mines records also permitted study to be made of the spatial and temporal distribution of the above 4 cancers. The results of the survey are in the main confirmed by those

FIG. 4.—Significant divergence of case numbers observed from those expected for oesophageal cancer in the Transkei.

found in the territories of origin of the miners. Thus, almost 70% of the cases of primary liver cancer came from Mozambique, which has the highest known incidence rate for this cancer in the world (Prates and Torres, 1965). Sixty-seven per cent of the cases of oesophageal cancer came from the contiguous areas of the Transkei and Ciskei, of which certain localities in the former have the second highest recorded rate in the world for oesophageal cancer (Doll, 1969). This cancer is rare in Mozambique with only 7 cases being found in a very large labour force over the period of 8 years. Whereas the ratio of oesophageal cancer to primary liver cancer in miners from Mozambique was 1 : 70, that in miners from the Transkei was 1 : 0·9, indicating that primary liver cancer was almost as frequent in miners' age groups in the Transkeian workers as oesophageal cancer.

At a local scale in Mozambique, higher case numbers of primary liver cancer were found in distinct localities in eastern coastal areas while lower case numbers were found inland, south and westwards. This pattern closely resembles that found in a hospital survey in southern Mozambique carried out by Purchase and Gonçalves (1971). At a local scale in the Transkei, significantly higher case numbers of oesophageal cancer were found in the south-west and centre, decreasing to the lowest case numbers in Pondoland in the north-east. This pattern closely resembles the incidence gradient found for males and females in the general resident

TABLE VII.—*Temporal Change of Crude Rates of Primary Liver Cancer and Oesophageal Cancer in African Gold Miners from Different Home Areas*

| | Liver | | | | | | Oesophagus | | | | | |
|---|---|---|---|---|---|---|---|---|---|---|---|---|
| | All miners | | | Mozambique | | | All miners | | | Transkei and Ciskei | | |
| Year | Mining population (man-years) | No. of cases | Crude rate | Mining population (man-years) | No. of cases | Crude rate | Mining population (man-years) | No. of cases | Crude rate | Mining population (man-years) | No. of cases | Crude rate |
| 1964 | 374455 | 95 | 25·4 | 77052 | 62 | 80·5 | 374455 | 17 | 4·5 | 107527 | 11 | 10·2 |
| 1965 | 369161 | 93 | 25·2 | 84904 | 62 | 73·0 | 369161 | 35 | 9·5 | 104603 | 24 | 22·9 |
| 1966 | 363232 | 92 | 25·3 | 88771 | 69 | 77·7 | 363232 | 27 | 7·4 | 98669 | 17 | 17·2 |
| 1967 | 353198 | 113 | 32·0 | 91792 | 79 | 86·1 | 353198 | 20 | 5·7 | 97308 | 13 | 13·4 |
| 1968 | 360732 | 95 | 26·3 | 90576 | 66 | 72·9 | 360732 | 22 | 6·1 | 100824 | 17 | 16·9 |
| 1969 | 355802 | 68 | 19·1 | 88346 | 47 | 53·2 | 355802 | 10 | 2·8 | 96977 | 7 | 7·2 |
| 1970 | 373504 | 85 | 22·8 | 92648 | 59 | 63·7 | 373504 | 12 | 3·2 | 85666 | 10 | 11·7 |
| 1971 | 376377 | 69 | 18·3 | 95898 | 43 | 44·8 | 376377 | 19 | 5·0 | 71175 | 10 | 14·0 |
| 8-year total | 2926461 | 710 | 24·3 | 709987 | 487 | 68·6 | 2926461 | 162 | 5·5 | 762749 | 109 | 14·3 |

population of the Transkei itself (Rose and McGlashan, 1975).

Another interesting feature is the close similarity of rates for all 4 cancers in both the Transkei and Ciskei, suggesting that these contiguous and ethnically similar areas are under uniform environmental risk.

In view of the very uneven geographical distribution of the cancer cases, it seems clear that mining operations have little or nothing to do with the induction of the cancers which appear to exist in an occult form by the time the African reaches the mine. Recruits seem to retain the original effects imposed upon them by their home environment, even to a strictly local scale within their homelands. This suggests that the recruits come into mine employment with the "imprint" of the site of the predominating cancer of their home areas already upon them, even at the comparatively early age at which they are recruited. This could mean that, for these 4 cancers at least, certain environmental risks, such as childhood exposure, diet or other social factors, are causative.

A steep drop in the crude incidence rate of primary liver cancer in mine workers from Mozambique was found from 1964 to 1971. The reason for this is unknown, but does not appear to be due to extraneous factors such as improvement in medical screening, efficiency of diagnosis or any change in recruiting policy. It is possible that some basic change in living conditions may have taken place in the fairly recent past which could have improved the environment as a whole, with particular regard to diet and health. No other significant temporal changes were found in the survey.

The present paper has sought to define spatial and temporal patterns only and has not in any way considered aetiology. The information presented, however, should be a logical starting place for further enquiry regarding the causation of these cancers.

Finally, it is clear that the survey has been predictive both of the cancers prevalent in the areas from which the miners were recruited and also of any changes which have occurred over time. For this reason, it is felt that cancer patterns in the miners should be studied continuously.

We thank Mr C. P. S. Barnard (Chamber of Mines) and mine medical officers at mine hospitals for case records, Mr P. G. D. Pretorius, General Manager, Crown Mines Ltd for access to compound records and Mr J. A. Gemmill, (W.N.L.A.) Recruiting Organisations (N.R.C.) Limited, Johannesburg, for providing the invaluable medical data which Mrs B. Stroud compiled for analysis. Mrs D. Fitzgerald is thanked for much assistance with preparation of the manuscript.

### REFERENCES

AMBROSE, P. (1969) *Concepts in Geography II.* London. Longmans: p. 284.

ARMSTRONG, R. W. (1969) Standardised Class Intervals and Rate Computation in Statistical Maps of Mortality. *Ann. Ass. Am. Geogr.*, **59**, 382.

BERMAN, C. (1935) Malignant Disease in the Bantu. *S. Afr. J. med. Sci.*, **1**, 12.

BURRELL, R. J. W. (1957) Oesophageal Cancer in the Bantu. *S. Afr. med. J.*, **31**, 401.

CHOYNOWSKI, M. (1959) Maps Based on Probabilities. *J. Am. Stat. Ass.*, **54**, 385.

COETZEE, A. M. (1965) Valedictory Address, Ann. Gen. Mtg, 22 April. *Proc. Mine med. Off. Ass. S.A.*, **44**, 101.

DOLL, R. (1969) The Geographical Distribution of Cancer. *Br. J. Cancer*, **23**, 1.

DOLL, R., MUIR, C. S. & WATERHOUSE, J. A. H. Eds. (1970) *Cancer Incidence in Five Continents.* Vol. II. Geneva: UICC.

DOLL, R., PAYNE, P. M. & WATERHOUSE, J. A. H. Eds. (1966) *Cancer Incidence in Five Continents.* Vol. I. Geneva: UICC.

GEDDES, E. W. (1969*a*) The Effect of Cancer Chemotherapy and Radiotherapy on the Clinical Course and Survival Time of Bantu Patients with Primary Carcinoma of the Liver. Thesis presented to the Faculty of Medicine of the University of Pretoria for the degree of Doctor of Medicine. **2**, 2.

GEDDES, E. W. (1969*b*) *Ibid.*, **1**, 34.

GEDDES, E. W. (1972) The Work of the Primary Liver Cancer Research Project: the Differential Diagnosis of Primary Malignant Hepatoma in Patients referred to the Liver Cancer Unit. *Proc. Mine med. Off. Ass. S.A.*, **52**, 25.

GEDDES, E. W. & FALKSON, G. (1973) Differential Diagnosis of Primary Malignant Hepatoma in 569 Bantu Mineworkers. *Cancer, N.Y.*, **31**, 1216.

HARINGTON, J. S. & MCGLASHAN, N. D. (1973*a*) The Temporal and Spatial Distribution of Liver

Cancer in African Gold Miners from Southern Africa. In *Liver*. Eds. S. J. Saunders & J. Terblanche. *Proc. Int. Liver Conf.*, Cape Town, Jan. 22–26, 1973, London : Pitman. p. 306.

HARINGTON, J. S. & McGLASHAN, N. D. (1973*b*) The Temporal and Spatial Distribution of Oesophageal Cancer among Mineworkers in Southern Africa. *Br. J. Cancer*, **28**, 86.

McGLASHAN, N. D. (1972) Ed. *Medical Geography Techniques and Field Studies*. London: Methuen. p. 11.

McGLASHAN, N. D. (1974) On the Reality of Spatial Variations of Morbidity and Mortality. *S. Afr. med. J.*, **48**, 1621.

PRATES, M. D. & TORRES, F. O. (1965) A Cancer Survey in Lourenço Marques, Portuguese East Africa. *J. natn. Cancer Inst.*, **35**, 729.

PURCHASE, I. F. H. & GONCALVES, T. (1971) In *Mycotoxins in Human Health*. Ed. I. F. H. Purchase. Proc. Symp. Pretoria, Sept. 2–4, 1970. S. A. Med. Res. Council and C.S.I.R., London: Macmillan. p. 263.

PURVES, L. R., MANSO, C. & TORRES, F. O. (1973) Serum α-fetoprotein Levels in People Susceptible to Primary Liver Cancer in Southern Africa. *Gann Monog. Cancer Res.*, **14**, 51.

ROBERTSON, M. A., HARINGTON, J. S. & BRADSHAW, E. (1971*a*) The Cancer Pattern in African Gold Miners. *Br. J. Cancer*, **25**, 395.

ROBERTSON, M. A., HARINGTON, J. S. & BRADSHAW, E. (1971*b*) The Cancer Pattern in Africans at Baragwanath Hospital, Johannesburg. *Br. J. Cancer*, **25**, 377.

ROSE, E. F. (1969) The Interplay of Factors Determining a Cancer Pattern. *Prog. exp. Tumor Res.*, **12**, 95.

ROSE, E. F. & McGLASHAN, N. D. (1975) The Spatial Distribution of Oesophageal Carcinoma in the Transkei, South Africa. *Br. J. Cancer*, **31**, 197.

SCHONLAND, M. & BRADSHAW, E. (1968) Cancer in the Natal African and Indian 1964–66. *Int. J. Cancer*, **3**, 304.

VON ZEYNEK, E. R. (1973) Survey of Cancer of the Oesophagus in Relation to Other Malignant Neoplasms. *S. Afr. med. J.*, **47**, 325.

WARWICK, G. P. & HARINGTON, J. S. (1973) Some Aspects of the Epidemiology and Etiology of Esophageal Cancer with Particular Emphasis on the Transkei, South Africa. *Adv. Cancer Res.*, **17**, 81.

WILSON, F. (1972) *Labour in the South African Gold Mines* 1911–69. African Studies Series 6, Cambridge: University Press.